# Molecular Mechanisms of Cutaneous Squamous Cell Carcinoma

**DOI:** 10.3390/ijms23073478

**Published:** 2022-03-23

**Authors:** Matthew L. Hedberg, Corbett T. Berry, Ata S. Moshiri, Yan Xiang, Christopher J. Yeh, Cem Attilasoy, Brian C. Capell, John T. Seykora

**Affiliations:** Abramson Cancer Center, CAMB Graduate Group, Department of Dermatology, Cutaneous Phenomics and Transcriptomics Core, Penn SBDRC, Perelman School of Medicine, University of Pennsylvania, Room 1011 BRB II/III, 421 Curie Blvd, Philadelphia, PA 19104, USA; matthew.hedberg@pennmedicine.upenn.edu (M.L.H.); corbett.berry@pennmedicine.upenn.edu (C.T.B.); amoshiri@derm.washington.edu (A.S.M.); yan.xiang@pennmedicine.upenn.edu (Y.X.); cjyeh@me.com (C.J.Y.); catill@comcast.net (C.A.); brian.capell@pennmedicine.upenn.edu (B.C.C.)

**Keywords:** skin cancer, squamous cell carcinoma, genetics of cancer, cancer biology

## Abstract

Non-melanoma skin cancers are cutaneous malignancies representing the most common form of cancer in the United States. They are comprised predominantly of basal cell carcinomas and squamous cell carcinomas (cSCC). The incidence of cSCC is increasing, resulting in substantial morbidity and ever higher treatment costs; currently in excess of one billion dollars, per annum. Here, we review research defining the molecular basis and development of cSCC that aims to provide new insights into pathogenesis and drive the development of novel, cost and morbidity saving therapies.

## 1. Introduction

Cutaneous squamous cell carcinoma (cSCC) is the second most common cancer among Caucasians, with estimates of approximately 1.1 million new cases annually in the US [1,2]. While outcomes are favorable for most patients, and the vast majority of cSCCs are cured with complete excision, an estimated 3–7% of patients develop metastases that lead to significant morbidity and mortality [2,3,4]. Thus, early diagnosis and treatment of cSCCs, and their premalignant precursors, is crucial to minimize morbidity and conserve healthcare resources.

Most cSCCs arise in a progressive fashion from premalignant/noninvasive precursor lesions [5]. The earliest clinically detectable precursor lesion is actinic keratosis (AK). AKs can be distinguished from surrounding keratinocytes by the presence of hyperplasia and hyperkeratosis clinically, and histologically by the presence of basal keratinocyte dysplasia and overlying parakeratosis [6]. AKs share some histologic findings with other, less common lesions that may themselves be precursors to keratinocytic malignancy, such as acanthosis, which is associated with human papillomavirus infection or arsenic poisoning. The most well studied and broadly accepted precursor lesion in the field cancerization theory, however, is the AK, which may persist as premalignant lesions, or even regress spontaneously [7]. A small percentage of AKs, incidentally, acquire additional genetic and epigenetic changes and progress to cutaneous squamous cell carcinoma in situ (SCCIS) and ultimately cSCC, both of which are clinically larger lesions, similarly characterized histologically by the presence of parakeratosis, with more pronounced dysplasia wherein the full thickness of the epidermis is replaced by malignant keratinocytes that are either bounded by the basement membrane in the case of SCCIS, or invade the dermis and surrounding structures in the case of cSCC [5]. A small subset of cSCC may acquire additional genetic and epigenetic features that lead to metastatic disease [8,9].

The evolution of malignant properties that underlies this progression is a current focus of research and encompasses the concept of field cancerization [10,11], which proposes that precancerous lesions such as AKs and SCCIS arise from mutated, subclinical clones of keratinocytes within a clinically unremarkable epidermis. In most instances, UV-exposure initiates the mutagenic process in skin, causing mutations in individual keratinocytes that, provided they confer a survival advantage, are selected over time. This selection results in the presence of multiple, mutated keratinocyte subclones within clinically normal appearing skin. Additional genetic and epigenetic changes in these clones can promote neoplastic selection to produce AKs, which can then progress to SCCIS and beyond, ultimately resulting in polyclonal cSCCs that are comprised of multiple, competing keratinocyte clones.

Molecularly, cSCC arises from the accumulation of genetic and epigenetic alterations in keratinocytes over time that permit development of an invasive tumor [9]. The accumulation of genetic alterations, and development of malignant and premalignant lesions, is accelerated when intrinsic defenses are compromised. Examples include patients on chronic immunosuppression, or those with a heritable predisposition to cancer, such as the disease states of Xeroderma Pigmentosum or Bloom syndrome [12,13]. DNA mutations causing qualitative changes in gene expression can occur due to defects in DNA replication, repair, or recombination mechanisms. Endogenous mutagens result in spontaneous alterations of DNA, including free radical damage due to reactive oxygen species, deamination, or depurination [14,15]. Exogenous mutagens include sunlight (UVB and UVA), smoking, and dietary components. Epigenetic alterations, leading to quantitative changes in gene expression, can also facilitate inappropriate transcriptional activation and silencing of genes [16]. Both genetic and epigenetic alterations can increase the overall mutation rate, enhance proliferation, and decrease cell death.

Advances in our understanding of the epidemiologic risk factors and molecular mechanisms driving tumorigenesis have resulted in new therapeutic options of varying efficacy for patients with locally advanced or metastatic disease [11]. The prevention of such disease all together rests upon the development of field treatments that largely target early-stage precursor lesions and subclinical disease. Currently, most field treatment protocols are hampered by poor compliance due to prolonged and repeated treatment periods, as well as clinical toxicity; their efficacy is difficult to evaluate in the absence of universally accepted endpoints and objective measurements of field disease [10]. These circumstances motivate research to identify new molecular targets for field treatment strategies in the early stages of disease to prevent progression to invasive, late-stage disease, with its inherent risk of metastases. In this review, we discuss recent findings on the genetics, epigenetics, and biology of cSCC development. The therapeutic implications of these data will also be discussed.

## 2. Genetic Alterations in cSCC

### 2.1. Mechanisms of Genetic Alterations in cSCC

Mutations in DNA can occur through a variety of mechanisms. DNA damage due to exogenous factors including UV light, chemicals, and ionizing radiation can lead to mutations if not repaired [15]. Mutations can also occur through endogenous factors such as mitotic errors, errors in DNA repair, or genome editing, as well as reactive oxygen species [14]. For cSCC to arise, the mutations need to occur in long-lived cells in the epidermal compartment which reside in the basal cell layer. DNA mutations that alter/destabilize the double helix structure, such as UV-induced pyrimidine dimers, are typically corrected through DNA repair processes such as nucleotide excision repair (NER). The importance of this evolutionarily-conserved mechanism is demonstrated by patients with Xeroderma Pigmentosum (XP) [17]. Loss of any one effector protein of the NER pathway leads to early development of premalignant lesions and malignant skin cancers [17,18,19]. While numerous mechanisms contribute to mutagenesis, epidemiological and clinical data support the concept that cumulative lifetime exposure to ultraviolet radiation (UV) is the primary carcinogen responsible for cSCC [20].

UV irradiation is capable of initiating and promoting the progression of all stages of squamous carcinogenesis [18,21]. Both UVB and UVA radiation promote skin cancer by altering keratinocyte signaling, inducing oxidative stress, and producing DNA mutations [22]. UV exposure leads to ATP consumption due to the activation of DNA repair systems, which when overactivated (e.g., PARPs) is detrimental for the cell [23] (PMID: 34638427). UVB radiation possesses sufficient photonic energy to promote structural rearrangements (DNA damage) resulting in cyclobutane pyrimidine dimers (CPD) or (6-4) photoproducts [23,24]. If the damaged DNA strand is not repaired prior to replication, the daughter strand acquires the change in base (e.g., C ≥ T) and a mutation has occurred [25]. This process generates a “UVB signature” characterized by high rates of C > T transitions and CC > TT double base changes [21,26]. UVA and UVB can produce cellular oxidative stress, leading to formation of reactive oxygen species (ROS); these compounds can promote 8-oxo-deoxyguanine formation, which, if not repaired prior to DNA replication, will lead to a G > A mutation. The location of these mutations and the resulting changes in the gene products and genome structure determines the fate of mutated keratinocytes.

### 2.2. Genomic Characterization of cSCC

Genomic analyses of cSCCs have revealed much about the molecular mechanisms that drive the transition from benign to malignant keratinocytes. cSCCs have the highest tumor mutation burden among malignancies harboring, on average, ≈50 mutations per megabase pair (Mbp) of DNA [15,26,27,28]. It is hypothesized that most of these mutations are “passengers”, offering little-to-no growth advantage nor impact on tumor progression, while a subset represent “driver mutations” that promote tumorigenesis by regulating cell fate, growth, survival, or genomic maintenance [9]. Whole exome and targeted sequencing analyses of unremarkable skin, AKs, SCCIS, and cSCCs provide evidence that numerous driver pathways act preferentially at specific stages to promote tumorigenesis [20,29,30,31,32,33,34]. Some of the mutated driver genes include *NOTCH1-3*,* TP53*,* FAT1*,* PIK3CA*,* CDKN2A*,* HRAS*,* KMT2C*,* KNSTRN*,* EGFR*,* CARD11*,* MYC*,* MLL2*,* MAP3K9*,* PTEN*,* SF3B1*,* VPS41*, and *WHSCI* [20,26,31,32,33,34,35,36]. However, our understanding of the molecular mechanisms and genetic drivers of cSCCs are complicated by the relatively high (~5.8 mutations per Mb) burden of somatic mutations in normal sun-exposed skin [26,37]. This mutation rate is similar to that observed in many cancers [15], indicating that normal sun exposed skin is a patchwork of precancerous cells harboring mutations in known driver pathways. Some of the most frequently cited genes mutated in normal appearing skin include known drivers such as *TP53, NOTCH1, NOTCH2*, and *FAT1* [34,37]. These findings have important implications for interpreting and understanding squamous carcinogenesis and suggest that certain mutations are somehow tolerated in vivo. Here, we will discuss the genetic alterations that have been repeatedly shown to play a role in cSCC tumorigenesis.

#### 2.2.1. TP53

*TP53* is the most commonly mutated gene in human cancers and plays a critical role in cSCC tumorigenesis [38]. The *TP53* tumor suppressor gene encodes a 53-kDa phosphoprotein that functions to regulate cell cycle arrest, senescence, DNA-repair, and apoptosis in response to cellular stress [39,40,41,42]. p53 transmits stress-inducing signals to different anti-proliferative cellular responses primarily by regulating transcription [43]. In an inactive state, p53 is bound to Mdm2, which antagonizes the transcriptional activity of p53, induces its ubiquitylation, and promotes nuclear export of p53 [43,44]. Traditionally, p53 activation in response to cellular stress (e.g., UV-induced DNA damage) occurs through stabilization of p53, sequence specific DNA binding, and transcriptional activation of target genes [45]. Stabilization of p53 occurs primarily through disruption of Mdm2 binding, which can occur through post-translational modification of the amino terminus of p53 or through direct regulation of Mdm2 [46,47]. One such regulator is p14^ARF^, a tumor suppressor transcribed from the *CDKN2A* gene, which directly binds to and inhibits Mdm2 to promote p53 activation [47,48,49]. Once stabilized, p53 binds to target genes (e.g., *CDKN1A* [50], *BAX* [51], and *DDB2* [52]) in a sequence-specific manner [53], activating or repressing gene expression [43]. p53 also transcriptionally regulates Notch genes in keratinocytes through a c-Jun dependent mechanism [54], and decreased p53 transcriptional activity correlates with lower levels of Notch-gene transcripts and protein [55].

Disruption of p53 activity can occur directly through inherited or acquired mutations in *TP53* or indirectly through dysregulation of p53 signaling. For example, *CDKN2A* mutations occur frequently in AK and cSCC and certain mutations have been shown to disrupt p53-dependent functions (discussed below). Mutations in *TP53* most frequently involve somatic deletions or missense mutations that alter p53 function [56]. Missense mutations in *TP53* most frequently involve 6 “hotspot” amino acids within the DNA binding domain [41,57,58,59]. Multiple mechanisms of action have been ascribed to various *TP53* alterations. Deletions and some mutations simply result in loss of function (LOF) of this important tumor suppressor. Other mutations inhibit p53 function by causing mutant p53 to act as a dominant negative inhibitor over WT p53 proteins [60]. Still, other missense mutations appear to confer gain of function (GOF) characteristics by altering the ability of mutant p53 to bind canonical p53 DNA binding regions and conferring the ability to bind alternative genes [61] and transcription factors (e.g., p63) [59]. GOF *TP53* mutations have been observed in subsets of patients with missense germline *TP53* mutations that have detectable mutant p53 protein expression and a significantly earlier cancer onset than patients with germline *TP53* mutations, resulting in a loss of p53 protein expression [59,62]. GOF *TP53* mutations have also been associated with an increased incidence of metastatic dissemination and drug resistance [63,64,65]. These findings indicate that malfunction of p53 is an important cause of carcinogenesis with implications for understanding cutaneous squamous cell carcinogenesis.

Analysis of mutations in *TP53* has established a clear connection between UV exposure, DNA damage, and skin carcinogenesis [41]. Early sequencing studies of human cSCC identified an abundance of point mutations in *TP53* resulting from UVB-induced C > T or CC > TT base substitutions [21]. In human sequencing studies, *TP53* mutations have been identified in approximately 60% of actinic keratoses and 50–90% of cSCC [36,66]. Studies in mice have shown that inactivating mutations in *TP53* promote AK and carcinoma in in situ formation [67,68]. In human cSCC, loss of p53 function has been shown to promote survival of mutant clones and the acquisition of additional mutations. Using whole exome sequencing, it was found that loss of the second *TP53* allele in human cSCCs strongly increases the likelihood of additional DNA mutations and chromosomal aberrations [69]. GOF mutations have also recently been described to promote HNSCC tumorigenesis, invasion, and metastasis through transcription-independent control of the p53-AMPK-FOXO3a-FOXM1 cascade [70].

While the role of *TP53* mutations in cSCC and its progression are clearly established, our understanding of the stage at which these mutations contribute most to cSCC carcinogenesis continues to evolve. Sequencing studies of grossly unremarkable sun-exposed skin identified clonal *TP53* mutations in seemingly normal skin [15,37,71]. AKs are collections of atypical keratinocytes, localized to the basal layer of the interfollicular epidermis and have the potential to progress to higher grade lesions via lateral and superficial expansion [72]. Recent sequencing studies of AKs, confirmed by microscopic examination, found *TP53* to be significantly mutated in AKs [33]. Another recent study, using laser capture microdissection of SCCIS and adjacent sun-exposed epithelium without microscopic evidence of dysplasia similarly found *TP53* to be mutated in SCCIS, but did not identify *TP53* mutations in microscopically normal skin [34]. These more recent studies that used microscopically confirmed lesional and non-lesional skin suggest that *TP53* mutations arise as early mutations in cSCC carcinogenesis and are enriched at the AK/SCCIS stage, compared to UV-exposed, histologically unremarkable skin [73]. Quantifying the mutational burden of *TP53* in “normal” skin is likely fraught with selection bias, as large samples of grossly normal skin likely contain microscopic collections of abnormal keratinocytes which may have gone on to form clinically discernable AKs capable of progression or regression.

Though *TP53* alterations clearly contribute to cSCC, they are not in and of themselves sufficient to establish or maintain the malignancy. Up to 60% of cSCCs are believed to arise from AKs, approximately 30% of AKs will spontaneously regress in one year, and the estimated annual rate of malignant transformation to cSCC per AK is 0.025–16%; with most studies estimating it at <1% [74,75]. Therefore, while mutation in *TP53* appears to occur early in the progression from normal to precancerous skin, and such mutations are selected for during malignant progression, additional mutations are needed to promote progression of precancerous lesion to cSCC. Further study of oncogenic *TP53* mutations in the early stages of UV-induced skin cancer is warranted.

#### 2.2.2. NOTCH

LOF mutations in *NOTCH1*, *NOTCH2* and *NOTCH3* have been found to occur commonly in cSCC and in sun exposed skin, including skin that shows no microscopic evidence of dysplasia [27,33,34,69,76,77]. *NOTCH1–3* genes encode Notch proteins that function in keratinocytes to regulate cell fate determination, stem cell potential, lineage commitment, proliferation, and survival [78]. Activation of Notch signal transduction occurs through binding of ligands expressed on neighboring keratinocytes. Notch1 receptors are expressed in all layers of the epidermis, while Notch2 is primarily expressed in the basal layer. The ligands expressed in the epidermis include Jagged-1 and Jagged-2, which are expressed predominately in the suprabasal layers and Delta-like 1, which is expressed throughout the basal layer [79]. Ligand receptor interaction between two neighboring cells results in proteolytic cleavage of the Notch receptor, nuclear translocation of the cleaved cytoplasmic domain, and subsequent binding to a downstream transcription factor CBF1, which thereby activates transcription [80].

Notch signaling plays a critical role in the development and maintenance of the epidermis [81]. In the suprabasal layers of the epidermis, increased Notch1 activation either directly or indirectly, through IRF6-dependent mechanisms, acts to suppress *P63* expression [82,83]. The shift from predominantly p63 to Notch signaling promotes cell cycle arrest and terminal differentiation. In addition, Notch signaling acts to promote keratinocyte differentiation through activation of caspase-3 and PKC-δ [84]. *NOTCH1* is a direct target gene of p53 and is downregulated in keratinocytes with *TP53* mutations [85].

Given its critical regulatory role in keratinocyte differentiation, it is not surprising that defects in Notch signaling are implicated in the pathogenesis of cSCC. Genetic loss of *NOTCH* promotes increased susceptibility to both chemically-induced and spontaneous cSCCs in mice, implicating Notch as a tumor suppressor [55,78,86,87,88]. Consistent with these findings, UV-signature LOF mutations in *NOTCH1* and *NOTCH2* have been found in approximately 75% of human cSCCs [27,77]. Recent studies indicate that UV-signature LOF *NOTCH* mutations also appear in clinically and microscopically non-dysplastic skin, suggesting that loss of Notch signaling may be one of the earliest mediators of transition from normal keratinocytes to a precancerous state [15,34,37,73].

Decreased Notch signaling may promote expansion of mutant keratinocytes in the epidermis via inhibition of UV-induced apoptosis of epidermal keratinocytes [89]. As a downstream effector of p53, *NOTCH1* is further implicated as a significant regulator in UV-induced skin cancer [55,85]. Notch and *TP53* alterations are not always mutually exclusive in cSCC and precancerous lesions, but the independent and coexistent alterations that have been reported vary, and further research into the relationship these alterations have with each other, and the progression of disease, is needed.

#### 2.2.3. RAS

Mutant Ras has been identified to act as a driver oncogene in an estimated one third of human cancers [90]. The canonical members (*HRAS*, *NRAS*, *KRAS4A*, and *KRAS4B)* and noncanonical members of the Ras superfamily are small guanosine triphosphatases (GTPases) that play a central role in transmission of mitogenic signals [90,91]. Ras GTPases cycle between GDP-bound inactive and GTP-bound active states [92]. In an active state, Ras-GTP binds to and activates a spectrum of downstream effectors to regulate signaling networks that control gene expression, proliferation, differentiation, and survival.

In cSCCs, reported rates of amplifications and activating mutations in *Ras* genes range widely from 3–30% [93,94,95]. *HRAS* mutations are more common than *NRAS* or *KRAS* mutations; characteristically occurring in codons 12, 13, and 61, at UV-sensitive CC sites [96,97]. In one study, 13/21 cSCCs (60%) that developed in patients on vemurafenib had *Ras* mutations; with 11/13 occurring in *HRAS*, exhibiting a significantly higher frequency of Ras mutations than is seen in cSCC outside treatment with B-Raf inhibitors [93,94,95,98]. This phenomenon is thought to occur because B-Raf inhibition leads to paradoxical activation of MAPK signaling. Driving accelerated growth of subclones containing *HRAS* mutations. Such activating *Ras* mutations have demonstrated oncogenic capacity in mouse models of chemically-induced keratinocyte papillomatosis [99]. However, in human patients with UV-induced DNA damage, GOF *Ras* alterations are exceedingly rare in AKs/SCCIS, and are encountered predominantly in a small subset of late-stage cSCCs [33,34,73]. As such, *Ras* genes are not generally regarded as oncogenes driving the early stages of cSCC development [9].

#### 2.2.4. CDKN2A

*CDKN2A* is inactivated in many human cancers and in families with hereditary melanoma and pancreatic cancer through homozygous deletion, methylation, or point mutation [100]. *CDKN2A* encodes two distinct tumor-suppressor proteins, p16^INK4a^ and p14^ARF^ that indirectly control the activities of p53 and retinoblastoma protein (RB) [47]. These two distinct proteins are derived from alternative transcription of the first exon of the *CDKN2A* gene [101]. INK4a and ARF, so-called because it is transcribed from an alternative reading frame (ARF), encode p16^INK4a^ and p14^ARF^, respectively. P16^INK4a^ protein directly inhibits the cyclin D-dependent kinases, CDK4 and CDK6, maintaining RB in its active, hypophosphorylated, anti-proliferative state [44]. While p14^ARF^ acts primarily as a negative regulator of Mdm2 [48]. Blocking its interaction with p53 by isolating Mdm2 within the nucleolus, and by inhibiting its E3 ubiquitin protein ligase activity [102,103]. Thus, both proteins are involved in modulating responses to hyperproliferative signals via RB and p53 transcriptional programs.

Mutations in the *CDKN2A* locus have been reported to occur in 24–45% of sporadic cSCCs [20,27,104,105]. About half these mutations affect both p16^INKa^ and p14^ARF^ [101]. A similarly high frequency of *CDKN2A* mutations has also been observed in small studies of metastatic cSCCs [106]. Moreover, previous comparative studies of AKs and cSCCs suggest that deletion of p16INKa increases the incidence of progression from AK to cSCC [107]. Combined with evidence that *CDKN2A* mutations are rare and are not found to be under positive selection in normal skin, altered function of p16^INKa^ and/or p14^ARF^ likely play an important role in the development of precancerous lesions, as well as the progression from precancerous to cancerous lesions [33,73].

#### 2.2.5. PIK3CA

A recent study of AKs and cSCCs found *PIK3CA* alterations, copy number amplifications, and/or GOF hotspot mutations in approximately half the sequenced samples in their cohort [33]. If these rates of alteration are confirmed in larger cohorts, they could be actionable therapeutic targets in cSCC. Similar alterations have been found in over one third of squamous cell carcinomas of the head and neck; where they have been shown to drive oncogenesis, along with increased sensitivity to targeted inhibitors, as well as show improved survival with nonsteroidal anti-inflammatory drugs [108,109]. Similar findings have been reported in colorectal and breast cancers [110]. This potential cross-cancer target could represent the next generation of application(s) for topical use tyrosine kinase inhibitors. An area of current research characterized by the December 2020 Food and Drug Administration approval of topical tirbanibulin, a Src kinase inhibitor with downstream activity against the phosphatidylinositol 3-kinase (PI3K) and other growth regulatory pathways, for the treatment of AKs.

#### 2.2.6. KNSTRN

*KNSTRN* encodes a kinetochore-associated protein that modulates anaphase onset and chromosome segregation during mitosis [35,111]. *KNSTRN* was the third most frequently mutated gene in a cohort of cSCC samples using a combination of single nucleotide variant (SNV) determinations from whole exome sequencing and targeted sequencing [35]. Over half of these mutations were mapped to aserine-to-phenylalanine mutations with C > T transition characteristic of UVB-induced mutagenesis. Subsequent mutational analyses identified *KNSTRN* mutations in the same functional domain but in a smaller proportion of cSCC samples [26]. *KNSTRN* mutations disrupt chromatid cohesion and correlate with increased aneuploidy in primary tumors and tumorigenesis in vivo [35]. The frequency of *KNSTRN* mutations has also been shown to correlate with progression of dysplasia from unremarkable epidermis to cSCC, suggesting that *KNSTRN* may play a role in early tumorigenesis of cSCC, though future studies will be needed [112].

#### 2.2.7. FAT1

*FAT1* encodes a tumor suppressor-related member of the FAT protocadherin family that is frequently mutated in numerous types of human cancers including cutaneous, head and neck, and oral SCCs [20,26,76,113,114,115]. However, *FAT1* mutations, including missense, deletion, and truncations have also been identified in a substantial subset of clinically unremarkable, sun-exposed skin samples [26,37]. The underlying functions of protocadherin proteins remain incompletely understood, especially in the skin. But two mechanisms that promote tumorigenesis have been reported; one in which inactivated *FAT1* acts as a tumor suppressor, resulting in aberrant Wnt/β-catenin signaling in multiple cancer types, and another in which loss of *FAT1* leads to increased *CDK6* expression via activation of the Hippo pathway [116,117]. Future studies will need to investigate the underlying mechanism that controls *FAT1* activity in normal, precancerous, and cancerous lesions.

#### 2.2.8. CARD11

Caspase recruitment domain 11 (*CARD11*) encodes a protein that acts as a scaffold for nuclear factor kappa B (NF-κB) activity [29,118]. Activating mutations in *CARD11* have been described in at least 2 independent studies suggesting a role for NF-κB signaling in carcinogenesis [29,119]. The NF-κB family of proteins consists of five transcription factors p65, RelB, c-Rel, NF-κB1, and NF-κB2 which form homo- and heterodimers that bind specific target genes to regulate transcription. In the epidermis, NF-κB plays a critical role in keratinocyte apoptosis, proliferation, and differentiation, but a more controversial role in tumorigenesis [120]. Findings from several studies point to a role of NF-κB in growth inhibition, as blockade of NF-κB activation induces spontaneous cSCC development in murine experimental models in part due to resulting *CDK4* upregulation [8,121,122]. However, in one study, mutations in the *CARD11* gene were found in 38% of 111 cSCCs with many of the mutations leading to constitutive NF-κB activity [119]. As has been seen with respect to *NOTCH1* and *TP53*, *CARD11* mutations have also been identified in sun-exposed and peri-tumoral skin, implicating aberrant NF-κB signaling in early steps of carcinogenesis. Thus, while changes in NF-κB-dependent gene expression have been shown to occur in cSCCs, additional studies are needed to clarify whether NF-κB plays a direct or indirect role in the progression to AK and/or cSCC.

#### 2.2.9. SRCASM

*SRCASM* (Src activating and signaling molecule, previously known as *TOM1L*) encodes a tumor suppressor platform molecule containing a VHS and GAT domain and multiple conserved tyrosine phosphorylation sites that are phosphorylated by activated Src-family tyrosine kinases (SFKs) [123]. When phosphorylated, Srcasm engages SFKs and downregulates their activity through a lysosomal-dependent mechanism. Functionally, this acts to limit keratinocyte proliferation and promote keratinocyte differentiation by dampening the activation of PDK1/Akt/mTOR, MEK/ERK, and STAT3 [55,123,124,125]. In support of a role for Srcasm in cSCC tumorigenesis, human SCCIS and SCCs have been shown to have decreased Srcasm levels and increased SFK activity relative to non-lesional tissue [55]. Furthermore, overexpression of Srcasm completely inhibits development of tumors in a murine model of cSCC in which constitutive activation of SFK protein Fyn in mice leads to spontaneous formation of precancerous lesions resembling AKs and cSCCs. Thus, Srcasm may act as an important tumor suppressor in human cSCCs and targeting SFK activity through modulation of Srcasm function may provide an important therapeutic strategy.

#### 2.2.10. TP63

While rarely mutated in human cancers, *TP63* is frequently overexpressed in lung, head and neck, and skin SCCs [126,127]. A homologue of the *TP53* gene, *TP63* encodes the p63 protein which plays a critical role in epithelial development and homeostasis [128,129]. *TP63*^−/−^ mice die shortly after birth, in part due to a complete lack of epidermis and epidermal structures [129]. *TP63* is transcribed from two different promoters giving rise to distinct transcription factors with (TAp63) and without (ΔNp63) the N-terminal p53-homologous transactivation domain [128]. These factors are also subject to alternative splicing resulting in α, β, and γ isoforms with different COOH-termini [130]. In the epidermis, ΔNp63α is the predominate isoform expressed in developmentally mature keratinocytes of the basal layer where it plays a critical role in maintaining proliferation potential and lineage specification [127,131].

Diverse transcriptional and post-transcriptional mechanisms regulate ΔNp63 activity. ΔNp63 can act as a dominant negative suppressor of the TAp63 isoform, thereby regulating p53 and TAp63 dependent gene expression [128,132]. A balance between the tumor suppressive functions of the TAp63 isoform and the oncogenic functions of the ΔNp63 isoform may play a critical role in proliferation and differentiation of stem cells and tumor cells [133]. ΔNp63α also acts to induce or repress a wide range of transcriptional targets through direct target gene binding, recruitment of epigenetic modulators/chromatin remodeling factors and non-coding RNA [134,135,136,137]. Crosstalk between p63 and Notch signaling plays a critical role in the balance between proliferation and terminal differentiation [127]. p63 functions as a selective modulator of Notch1 target gene expression (e.g., *HES1*) and also acts as a negative regulator of *NOTCH1* expression via p53 [83,138]. This serves to limit Notch signaling in the basal epidermis, which, together with p63′s suppression of the cyclin dependent kinase (CDK) inhibitor, p21^WAF1/Cip1^, maintains proliferation and inhibits terminal differentiation in the basal epidermis [83,127,139].

Consistent with a role in squamous cell carcinogenesis, forced overexpression of ΔNp63α in the basal layer of stratified epithelia is sufficient to induce mild epidermal hyperplasia and predisposes to papilloma formation in a murine chemical model of cSCC [140]. Genetic ablation of *p63* in established murine SCC tumors results in regression via reduced cell proliferation and increased apoptosis driven by reduced expression of the p63 target gene *FGFR2* [141]. ΔNp63α has also been shown to drive proliferation in SCC through suppression of *TGFB2* expression and *RHOA* activity [142]. While *TP63* amplification was reported to be present in a modest proportion of SCC, including 24% of metastatic cSCCs, additional mechanisms account for dysregulated ΔNp63 mRNA and protein expression in SCCs [29]. Nucleoporins (*NUP62*) have been shown to be important in the translocation of ΔNp63α to the nucleus in a ROCK-phosphorylation dependent manner [143]. Elevated levels of *NUP62* have been demonstrated in cSCC, while reduction of *NUP62* expression has been shown to prevent proliferation of SCC cells. Elevated ΔNp63 expression may also result from increased expression of Syntaxin binding protein 4 (Stxbp4), which acts to suppress the anaphase-promoting complex/cyclosome (APC/C) mediated ubiquitination and proteolysis of ΔNp63 [133,144]. Epigenetic dysregulation of the iASPP-P63 feedback loop at the microRNA level has also been shown promote proliferation and block epithelial-mesenchymal transition in cSCC [145]. Thus, through numerous regulatory pathways, *TP63* plays a key role in cSCC tumorigenesis.

#### 2.2.11. EGFR

Epidermal Growth Factor Receptor (*EGFR*) signaling is dysregulated in many human cancers, including cSCC. The EGFR is a transmembrane glycoprotein with numerous ligands (e.g., EGF, transforming growth factor alpha) that, upon binding, initiate homo- and hetero-dimerization, transphosphorylation, and subsequent activation of downstream pathways including Ras-Raf-MEK-ERK, PI3K, and JAK/STAT signaling [146]. Depending on the ligands present, receptor expression, and differentiation state, EGFR activation can regulate these signaling networks to influence keratinocyte proliferation, differentiation, and survival [147]. Normally, high EGFR activity acts in the basal epidermis to maintain self-renewal and suppress differentiation. In the upper layers, *EGFR* is downregulated to promote keratinocyte differentiation. Importantly, EGFR signaling acts to downregulate both *TP53* and *NOTCH1* transcription through a c-Jun dependent mechanism inhibiting keratinocyte differentiation and apoptosis [54].

Dysregulation of EGFR signaling can arise through *EGFR* mutations, receptor/ligand overexpression, and alterations in trafficking and signaling pathways [146]. As opposed to pulmonary SCC, *EGFR* is infrequently mutated in cSCC with reports ranging from 1–20% [148,149]. *EGFR* alterations were identified in only 6/122 (4.9%) recurrent or metastatic cSCCs, most of which (4/6), were amplifications [31]. Importantly, EGFR signaling is dysregulated in a much higher proportion of tumors than those predicted by genetic mutation alone. In one cohort of 94 cSCCs, *EGFR* was overexpressed, aberrantly localized and amplified in 35%, 53% and 7% of tumors, respectively [150]. Overexpression of *EGFR* may result from a combination of increased mRNA synthesis and decreased degradation. For example, *TP53* mutations have previously been shown to positively regulate *EGFR* expression levels [151]. *TP53* mutations in AK/SCCIS and cSCC may therefore promote enhanced EGFR signaling. Epigenetic regulation of *EGFR* expression may also play an important role in its overexpression. Long noncoding RNA (lncRNA) metastasis-associated lung adenocarcinoma transcript 1 (MALAT1) promotes *EGFR* expression in cSCC through a c-Myc and kinectin 1 dependent mechanism [152]. A recent study identified microRNA-27a (miR-27a) as a negative regulator of EGFR signaling whose expression was significantly downregulated in human cSCCs [153]. Collectively, these findings indicate that EGFR signaling is dysregulated through multiple mechanisms during cSCC tumorigenesis.

### 2.3. Transcriptional Characterization of cSCC

Numerous studies have used transcriptional analyses of AKs and cSCCs to define how changes in gene expression regulate tumorigenesis [115,154,155,156,157,158,159,160,161,162]. However, findings from these studies have been limited by confounding variables including high inter-patient/sample variability, lack of histologic confirmation of skin tissue, and high annotation error rates.

A recent, well-designed study used combined next-generation sequencing analyses of patient-matched, histologically validated “normal,” skin (NS), AK and cSCC samples and a UVR-driven mouse model to identify transcriptional drivers involved in cSCC development [26]. Whole exome, RNA-seq, and miRNA-seq experiments were performed on multiple samples from 9 patients. Whole exome sequencing (WES) identified many of the commonly reported significantly mutated genes (SMG) including *TP53*, *NOTCH1–2*, *FAT1*, *MLL2*, and *KNSTRN*, but interestingly found that AKs contained the greatest proportion of SMGs, suggesting that AKs have acquired the mutational events necessary for cSCC formation. Consistent with these findings, global gene expression patterns revealed that dysregulated gene expression occurs at the earliest transition from NS to AK versus the subsequent transition to cSCC. Using a transcription factor (TF) binding motif analysis, the authors then identified several putative TFs responsible for altered gene expression some of which included ETS2, SP1, FREAC2 (FOXF2), AP1, NFAT, TCF3, LEF1, E2F MYC, and NFY. ETS2 is a pro-oncogenic TF downstream of the ERK MAP kinase pathway with targets that were broadly upregulated [163]. TCF3 and LEF1 are both downstream of the B-catenin/Wnt signaling pathway whose activity was upregulated across most samples. NFAT/AP1 target genes were downregulated early, which the authors proposed may reflect inhibition of keratinocyte differentiation programs that may be modulated by NOTCH signaling. While these findings suggest that these TFs may serve as key transcriptional regulations that drive cSCC development, their role in tumorigenesis remains to be functionally validated.

## 3. Beyond the Genome: Epigenetic Regulations in cSCC

Epigenetic alterations represent additional hallmarks in a cell’s transformation into cancer. These alterations result in a loss of gene expression by transcriptional silencing, via epigenetic promoter hypermethylation of CpG islands. Epigenetic alterations are estimated to be about 10 times more common across all cancer types than genetic mutations [164]. Epigenetic regulation in cells is achieved through a variety of mechanisms including DNA methylation, histone modifications (methylation, acetylation, phosphorylation, ubiquitination, and sumoylation), chromatin remodeling, and microRNAs. Epigenetic dysregulation can result from alterations to any of these systems, such as overactivation of methyltransferases or histone methyltransferases (PMID: 23455543). Affording a wide range of potential therapeutic targets holding great promise for cancer prevention, detection, and therapy [165]. The nascent field of epigenetic alterations in cSCC continues to garner increasing attention.

### 3.1. Aberrant DNA Methylation in cSCC

Genomic DNA methylation alterations, such as global DNA hypomethylation and gene specific hyper- or hypomethylation, are associated with SCC. Examples include *CDKN2A* (p14^ARF^ and p16^INK4A^) promoter methylation, found in 40% of cSCC, and hypermethylation of the *FOXE1* promoter [166,167]. *FRZB*, an antagonist of Wnt signaling, was found to be differentially methylated in metastatic cSCC as compared to primary cSCCs [168]. E-cadherin is among the genes most frequently hypermethylated in SCC [169]. This adhesion molecule promotes the structural integrity of the epidermis, loss of expression secondary to methylation may facilitate malignant invasion. Promoter hypermethylation of E-cadherin in keratinocytes coincided with the acquisition of a metastatic phenotype in a chemical carcinogenesis mouse model of cSCC [170]. This same study identified a number of novel methylation targets, such as insulin-like growth factor binding protein-3, in malignant murine keratinocytes, which correlated with hypermethylation patterns seen in human primary cSCCs.

### 3.2. Histone Modifications in Cutaneous SCC

Posttranslational covalent modifications of histones include acetylation, methylation, phosphorylation, ADP-ribosylation, ubiquitination, sumoylation, arginine deamination, and proline isomerization, occurring primarily at the amino-terminal histone tails. The location and type of modification impact the effect on gene transcription. Synergistic cooperation between different histone modifications acts to either repress or activate transcription. The overarching epigenetic state of the cell is ultimately defined by the specific combination of histone modifications present across the chromatin. This “histone code” represents an epigenetic readout reflecting the transcriptional state of a DNA region [171].

Histone acetyltransferase p300 (coded by *EP300*) has been shown to be essential for cell-cycle withdrawal of terminally differentiating keratinocytes, via several transcriptional regulatory mechanisms [172,173]. Similarly, histone methyltransferase (*EZH2*), a key component of the Polycomb repressive complex 2, is another epigenetic regulator of squamous differentiation and importantly, represents a druggable target [174]. The frequently mutated *EZH2* regulates self-renewing keratinocyte populations through repression of *INK4A-INK4B*, preventing the recruitment of AP1 transcriptional factors to genes involved in terminal differentiation [175,176]. Sustained *EZH2* activity is required for survival of keratinocyte cancer stem cell populations [177]. Increased *EZH2* expression has been associated with malignant progression in both cutaneous and bronchial epithelium [178,179]. In HNSCC cells, *EZH2* promotes enhanced malignant progression by mediating the effects that increased levels of the long non-coding RNA *HOTAIR* have on E-cadherin expression [180].

### 3.3. Long Noncoding RNA Dysregulation in cSCC

Stretches of DNA between genes, called non-coding DNA, also harbor genetic variants that are associated with diseases such as cSCC. Long noncoding RNAs (lncRNAs) are untranslated RNA transcripts >200 nucleotides in length that can regulate gene expression. Several lncRNAs have been shown to play an important role in keratinocyte differentiation and cSCC pathogenesis, including *TINCR*, *PICSAR*, *LINC00520*, *LINC00319*, *MALAT1*, *LINC01048*, *GAS5*, and *HOTAIR* [181]. *TINCR* is upregulated during keratinocyte differentiation and downregulate in cSCC, suggesting a pro-differentiation/antineoplastic role in keratinocytes [182]. *PICSAR* is expressed preferentially in cSCC tumor cells and promotes ERK1/2 activation by downregulating dual specificity phosphatase 6 (*DUSP6*) promoting cell migration through regulation of integrin expression [183]. *LINC00520* expression is decreased in cSCC A431 cell lines and overexpression was shown to decrease the activity of EGFR and PI3K mRNA and protein in vitro [184]. *LINC00319* expression, which promotes proliferation and migration by regulating miR-1207-5p mediated regulation of cyclin-dependent kinase 3, is significantly increased in cSCC [185]. *MALAT1* promotes cSCC oncogenesis through regulation of EGFR activity [152]. A MALAT1-c-Myc complex binds the kinectin 1 (*KTN1*) promoter, enhancing its transactivation to positively regulate EGFR protein expression. *LINC01048* expression, which increases binding of TAF15 to the Yes-Associated Protein 1 (*YAP1)* promoter, is associated with increased mortality in cSCC [181,186]. *YAP1* encodes a key coactivator of Hippo signaling, which promotes RAS activation along with downstream AKT and ERK signaling [187]. *GAS5* is a tumor suppressor with decreased expression in cSCC and other cancers [188]. In cSCC cell lines, *HOTAIR* is overexpressed and acts with miR-326 to regulate *PRAF2* expression [189]. The network of lncRNA interactions that regulate cSCC pathogenesis is complex. Further validation and study of lncRNAs may present novel therapeutic opportunities in cSCC.

### 3.4. MicroRNA Dysregulation in cSCC

MicroRNAs (miRs) are short non-coding RNAs of 19–25 nucleotides that regulate gene expression post-transcriptionally. MiRs are involved in many biological processes and their dysregulation in cancer is believed to be important to tumorigenesis [190]. Nearly 20 oncogenic miRs have been identified (e.g., miR-21, miR-135b), along with tumor suppressor miRs (e.g., miR-124, miR-125b) in cSCC [191].

miR-21 is highly expressed in human cSCCs and HNSCCs. Where it inhibits *PTEN* and *GHRL3* expression, leading to enhanced PI3K/AKT/mTOR activity [192]. Downregulation of miR-203, an antagonist of p63, and expression of miR-21, is associated with metastasis in HNSCC [193]. miR-124 and miR-214 are both downregulated in cSCC and may drive overexpression of ERK1/2 which promotes cellular proliferation in cSCC [194]. miR-27a targets and inhibits *EGFR*, the downregulation of miR-27a correlates with upregulation of EGFR in cSCCs [153]. Overexpression of miR-365 inhibits expression of the negative regulator of HIF1-alpha, *HOXA9*, which functions as a tumor suppressor in human cSCCs [195,196].

miRs, including miR-125b and miR-135b, have also been associated with tumor cell invasion. The downregulation of miR-125b in early cSCC promotes tumor growth and motility through a network of pro-tumorigenic genes including matrix metalloproteinases *MMP13*, *MMP7*, and *MAP2K7* [197]. miR-135b is overexpressed in cSCC and promotes cancer cell motility and invasiveness [198].

TAp63 regulated miRs, miR-30c-2* and miR-497, act to inhibit tumor cell proliferation and promote apoptosis. Introduction of miR-30c-2* or miR-497 mimics massively inhibited growth of cSCC tumor xenografts in vivo [199], demonstrating that miR dysregulation in cSCC may contain opportunities for novel therapeutic development.

### 3.5. UV-Induced Epigenetic Regulation in cSCC

The primary environmental risk factor for BCC, SCC, and melanoma is ultraviolet radiation, which, like arsenic, acts at the molecular level, in part, through epigenetic mechanisms. Analysis of the epigenetic patterns of the tumor suppressor *P16INK4a* in chronically UVA-irradiated HaCaT human keratinocytes revealed a striking reduction of the permissive histone mark H3K4me3 in the promoter (4–9 fold reduction for 10 and 15 weeks, respectively), which has often been found deregulated in skin cancers [200]. This alteration, together with severe promoter hypermethylation, strongly impaired the transcription of *P16INK4a* (20–40 fold for 10 weeks and 15 weeks, respectively). Analysis of tumor cells also revealed downregulation of *P16INK4a* transcription, attributed to enrichment of the heterochromatin histone mark H3K9me3, the repressive mark H3K27me3 and promoter hypermethylation. Less pronounced UVA-induced epigenetic alterations were identified in other genes *KLF4* and *NANOG* (stem cell fate determination), h*TERT* (telomere maintenance), and *P21WAFI/CIPI* (tumor suppressor), demonstrating that UVA can alter skin cancer associated transcriptomes by means of epigenetic DNA and histone alterations.

### 3.6. Epigenetic Regulation in cSCC Metastasis

Epigenetic alterations have also been shown to contribute to the final stages of cSCC progression metastasis. The epigenetic profiles of metastatic cSCCs diverge from those of their corresponding primary lesions, including significantly increased hypermethylation at *FRZB* [168]. Epigenetic profiling may prove to be a prognostic biomarker in nonmelanoma skin cancers. Super enhancer and transcriptional profiling of stem cell populations isolated from keratinocyte-derived SCCs suggest the Ets2 transcription factor to be a key regulator of epigenetic changes associated with malignant behavior [201].

### 3.7. Discovery of Novel Epigenetic Biomarkers in cSCC

Our understanding of the importance of global genomic DNA methylation in the molecular pathogenesis of neoplasia is growing. DNA methylation events may represent a specific and early marker of tumorigenesis that can be easily detected by minimally invasive PCR based methods. Promoter DNA methylation gene panels exist for HNSCC screening, risk of recurrence, and assessment of margin status during surgery. No correlation between global histone modifications and prognosis has yet been described in cSCC though such correlations have been identified in esophageal SCC. Scientists are now generating extensive maps of histone modifications and DNA methylation across mammalian cell types utilizing high throughput sequencing technologies totaling over 400 currently known marks [202]. Deciphering the specific functions of each of these marks in regulating gene expression will enable research into many aspects of human health and disease.

### 3.8. CRISPR/Cas9 Tools of Epigenomic Editing

Utilizing the CRISPR/Cas9 bacterial antiviral system for scientific research has been transformative in biomedicine [203]. Major limitations to editing human DNA with the CRISPR/Cas9 system are off target effects, however. The engineered Cas9-KRAB fusion protein activates specific genes by targeting specific histones for modifications such as the acetylation-one type epigenetic mark. Repression mediated by Cas9-KRAB via modification of the epigenome at specific positions has been shown to specifically disrupt gene expression [204,205]. This novel system allows for direct functional analysis of site-specific epigenetic modifications and modulation of target genes, offering a powerful novel platform for research and therapeutics in cSCC.

## 4. Metabolic Reprogramming in cSCC

Metabolic reprogramming is a hallmark of many cancers, including skin cancer [206,207]. Notably, melanoma metabolism has been intensively studied, and its metabolic signaling pathways have been shown to be involved in cancer cell survival, invasion, metastasis, and resistance to BRAF inhibitor therapy or PD-1 blockade immunotherapy [208]. While our understanding of the role and mechanisms of metabolic reprogramming in non-melanoma skin cancer is somewhat limited, recent studies have highlighted its importance in cSCC carcinogenesis including the effects of the DNA damage response, the potential role of commonly mutated oncogenes and tumor suppressors, and the role of deletions in mitochondrial DNA (mtDNA).

The Warburg effect is a metabolic feature of many cancer cells in which cells use glycolytic pathways despite the presence of adequate oxygen supply [209]. This reprogramming from oxidative phosphorylation (OXPHOS) to glycolysis is thought to allow cancer cells to produce ATP at higher rates/levels than OXPHOS while also producing substrates for anabolic metabolism (e.g., lactate). This appears to hold true for cSCC as recent studies have identified that, relative to peritumoral skin, hyperplasia and AKs, human cSCCs express higher levels of glycolytic enzymes, and lower levels of enzymes involved in lipid biosynthesis and the TCA cycle [210]. Murine models of cSCC utilizing oncogenic *Ras* mutation coupled with loss of *p53* in hair follicular stem cells (HFSC) similarly find that aerobic glycolysis is enhanced in cSCCs [211]. Targeting this metabolic shift may provide a path to selectively target neoplastic cells. Indeed, selective inhibition of GLUT1 in squamous cell lung cancer using WZB117, a small molecular GLUT1 inhibitor, reduced tumor growth of HCC1588 and HCC2814 cSCCs by 40% while adenocarcinoma H1299 tumor growth remained unaffected [212]. However, the mechanisms and the functional role of metabolic reprograming in cSCC initiation and progression remain controversial.

Many of the commonly mutated oncogenes and tumor suppressors implicated in cSCC carcinogenesis play an important role in regulating energy metabolism. For instance, p53 influences energy metabolism by enhancing OXPHOS and inhibiting glycolysis through several mechanisms [207]. *RAS* mutations in cancer cell lines are associated with metabolic reprogramming through regulation of glycolytic enzyme gene expression and glutamine metabolism [213,214,215]. *CDKN2A* mutations and deficiency of p16^INKa^ have been shown to regulate gluconeogenesis [216]. Furthermore, beyond somatic mutations, dysregulation of tumor suppressor and oncogene activity may influence cSCC carcinogenesis. In a recent study, Homeobox A9 (*HOXA9*), a direct target of onco-miR-365, was significantly downregulated in both cSCC primary tumors and cell lines. *HOXA9* acts as a tumor suppressor and inhibits glycolysis in cSCC in vitro and in vivo by negatively regulating HIF-1α and its downstream glycolytic regulators, *HK2*, *GLUT1* and *PDK1* [196]. While the specific role of commonly mutated or dysregulated oncogenes and tumor suppressors in metabolic reprogramming of cSCC remains to be determined, these findings demonstrate multiple mechanisms through which metabolism may be altered in cSCC.

In addition to somatic mutations in oncogenes and tumor suppressors, mutations in mtDNA, triggered by genomic instability or UV exposure, are implicated in metabolic reprogramming and cSCC pathogenesis [217,218,219]. mtDNA encodes 13 essential genes for mitochondrial OXPHOS along with 2 ribosomal RNAs and 22 transfer RNAs [220]. Mutations in these critical genes have been shown to result in mitochondrial dysfunction, altered ATP production, and are associated with carcinogenesis [220]. For example, mtDNA mutations associated with genomic instability from knockdown of the tumor suppressor *XPC* lead to decreased OXPHOS and increased glycolysis through an *AKT*, NAPDH oxidase-1 (*NOX1*), and reactive oxygen species (ROS) dependent mechanism [221,222]. Moreover, several common mutations in mtDNA have been identified in sun-exposed skin including a 260 bp mtDNA tandem duplication in the regulatory site of mitochondrial DNA (D-loop) [217], a 4977 bp deletion [217,219], and a 3895 bp deletion [223]. Additional studies are needed to determine the specific role that each mtDNA mutation plays in keratinocyte metabolism and cSCC carcinogenesis.

While cumulatively this work sheds light on the mechanisms controlling metabolic reprogramming in cSCC, how metabolic changes regulate carcinogenesis/tumorigenesis remains controversial. Some contend that cSCC may not require increased glycolytic activity for transformation [211]. Aerobic glycolysis culminates in NADH-dependent fermentation of pyruvate to lactate by lactase dehydrogenase (*Ldh*) and loss of *Ldha* activity dramatically reduces glycolysis [211]. Surprisingly, deletion of *Ldha* in a genetic (*Ras* and *p53*-null driven) and a chemically induced DMBA/TPA model of cSCC failed to cause any changes in tumorigenesis, including tumor number, time to formation, proliferation, volume, gene expression, and immune response. While *Ldha*-null tumors took up and used more glutamine, suggesting a possible compensatory mechanism, the results suggest that cSCC does not require increased glycolytic activity or reprogramming to generate cancers. Investigations into whether metabolic changes occur during the initial phase of carcinogenesis identified that UVB exposed keratinocytes undergo a metabolic shift in response to DNA damage leading to a downregulation of glycolysis, TCA cycle, and fatty acid β oxidation, and an upregulation of the electron transport chain (ETC) before overt tumor formation [210]. Overactivation of the ETC results from upregulation dihydroorotate dehydrogenase (*DHODH*) which acts to coordinate nucleotide biosynthesis. Notably, Leflunomide, a non-specific inhibitor of DHODH, blocks UVB-induced tumorigenic transformation of keratinocytes, raising the possibility of targeted metabolic therapy for the prevention of UVB-induced cSCCs [224]. These studies provide compelling evidence that metabolic reprogramming is robust in cSCC, but its exact role and the underlying mechanisms controlling these processes remain to be elucidated.

## 5. Current Status of Molecular Targeted Therapies in cSCC

Targeted therapies for basal cell carcinoma and melanoma were derived via elucidation of the molecular mechanisms driving their tumorigenesis. Mutations in the hedgehog pathway led to the application of smoothened inhibitors for the treatment of basal cell carcinoma and *BRAF* mutations have been successfully targeted with BRAF inhibitors in melanoma. Enhanced understanding of cSCC tumorigenesis and mechanisms driving formation of its precursor lesions may similarly drive the development of novel treatment strategies. Identification of key driver genes in cSCC has been hampered, in part, by the high mutational burden of cSCCs. However, EGFR inhibitors and tyrosine kinase inhibitors have shown varying degrees of efficacy in clinical trials and are promising areas of ongoing research alongside the continued development of other novel targeted therapies, including immune checkpoint inhibitors, which represent the only FDA approved targeted therapies in cSCC at this time.

### 5.1. EGFR Inhibitors

EGFR inhibitors, including small molecule inhibitors (e.g., gefitnib and erlotinib) and monoclonal antibodies (e.g., cetuximab and panitumumab) are well studied. The relatively high frequency of tumors with dysregulated EGFR signaling and the established safety/efficacy of these therapies in other malignancies makes them attractive for use in cSCC. EGFR small molecule inhibitors act by blocking the tyrosine kinase ATP binding site, thereby inhibiting downstream signal transduction, whereas monoclonal antibodies bind to the extracellular domain of EGFR inhibiting phosphorylation and activation [225]. In phase II trials for patients with incurable, recurrent, or metastatic cSCC, oral gefitinib and erlotinib had limited efficacy with overall response rates (ORR = partial response plus complete response) ranging from 10–16% [226,227]. Cetuximab, a chimeric mouse-human anti-EGFR IgG1 monoclonal antibody, has demonstrated improved ORR of 28% in the initial phase II trial [228] and as high as 42–53% in recent retrospective analyses [229]. Similarly, phase II studies with panitumumab, a high affinity human anti-EGFR IgG2 monoclonal antibody, demonstrated an ORR of 31% [230]. While the response rates to date have been somewhat limited, the development of predictive biomarkers to identify susceptible subpopulations and/or combination therapy with other targeted therapies and platinum-based chemotherapy may further improve response rates [231,232]. A limitation to the use of EGFR inhibitors is their propensity to cause skin reaction side effects: acneiform rash, pruritus, desquamation, hypertrichosis, and/or nail disorders often requiring treatment that can be seen in as many as 80% of treated patients, in the case of cetuximab [233]. The major challenge of systemic EGFR targeted therapies is that low response rates have been seen alongside expected, and notable, side effect profiles, arguing against the use of these agents in unselected cSCC patient populations, on balance. One relatively unexplored avenue is the application of topical EGFR inhibitors.

### 5.2. Immune Checkpoint Inhibitors

Immune checkpoint blockade has been employed across many malignancies including cSCC. Cemiplimab is a high-affinity, human hinge-stabilized IgG4 monoclonal antibody to the PD-1 receptor that acts to enhance responses of human primary T cells [234]. Following promising early results and aggregated data analysis of 108 patients with advanced (75 metastatic/33 locally advanced) cSCC, from a phase I/II clinical trial (R2810-ONC-1423) and phase II clinical trial (R2810-ONC-1540), which found a combined ORR of 47% (95% CI: 38, 57), with 4% complete and 44% partial response rates including durable responses of 6 months or more in 61% of responders, the FDA approved cemiplimab as the first targeted immunotherapy in cSCC on Sept 28, 2018 [235,236]. On 24 June 2020, the FDA approved pembrolizumab, an IgG4-kappa humanized monoclonal antibody that also targets the PD-1 receptor, following KEYNOTE-629 (NCT03284424); a multicenter, multi-cohort, non-randomized, open-label trial in which ORR was 34% (95% CI: 24, 44) and median response duration was not reached (range: 2.7, 13.1+ months) [237]. Additional phase II trials of pembrolizumab as a first-line single-drug therapy in unresectable cSCC have shown similar results [238]. Some evidence exists to suggest that cemiplimab may be superior to other systemic therapies in cSCC [239]. However, these represent indirect treatment comparisons and many trials with agents such as nivolumab (NCT04204837), as single agents and in combination with other systemic therapies, are ongoing, which may provide substantial benefit as has been suggested in isolated case reports to date [240,241]. One potentially significant limitation of immunotherapy in advanced cSCC is that advanced cSCC is most prevalent among solid organ transplant patients, where the use of such agents may increase the risk of allograft rejection [233].

### 5.3. SRC Family Kinase (SFK) Inhibitors

Both preclinical murine data and early phase clinical trials suggest targeting SFKs may be a promising avenue for the treatment of AKs and SCCs [242,243]. Dasatinib is a multi-kinase inhibitor that potently inhibits SFK activity, along with other kinases including BCR/ABL, c-KIT, ephrin family kinases, and PDGFR [244]. Studies in mice demonstrated that a topical formulation of dasatanib induced similar rates of regression in cSCCs versus topical 5-Fluorouracil (5-FU) without the epidermal ulceration or severe toxicity/death that has been seen with topical 5-FU use in mice [242]. Tirbanibulin (KX2-391) is a well-characterized SFK inhibitor used as a systemic agent in multiple cancer clinical trials, that also disrupts actin polymerization and microtubule formation at higher doses and has been FDA approved for topical use in AK following results of two multi-center trials in which tirbanibulin was shown to be superior to placebo at 2 months but showed high rates (47%) of local recurrence among complete responders at one year [245,246].

### 5.4. PI3K/mTOR Inhibitors

The PI3K and mammalian target of rapamycin (mTOR) pathways act as key regulators of a broad range of cellular functions, including cell growth, metabolism, survival, and differentiation [247]. Upregulation of PI3K/mTOR signaling is a frequent finding in SCC, especially in HNSCC, and there are several clinical trials underway to investigate PI3K/mTOR inhibitors for HNSCC [248,249]. In cSCC, the number of studies investigating PI3K/mTOR inhibitors is limited. One study found systemic BEZ-235, a PI3K/mTOR inhibitor, by oral gavage to be effective in inhibiting the formation of papillomas and cSCCs in a chemically induced mouse model of cSCC, but to be ineffective in treating established lesions in preclinical models derived from human cSCC cell lines [250]. A subsequent study using the K14-Fyn Y528 transgenic mouse model of cSCC, however, showed topical application of BEZ-235 in mice yielded similar results to dasatinib; efficient regression of cSCC with less inflammation, no ulceration, and no mortality compared to 5-FU [242]. LY3023414, an orally bioavailable PI3K/AKT/mTOR inhibitor, has been shown to have cytotoxic activity in vivo in cSCC tumor xenograft models [251]. A clinical trial of a topical mTOR/PI3K inhibitor called CLL442 in patients with cSCCIS found twice daily topical application was safe and well tolerated with no severe adverse events. However, the primary endpoint of lesion reduction or complete lesion clearance was not met (NCT03333694). Given the promising preclinical data and mounting evidence of the efficacy of PI3K/mTOR inhibitors in other cancer types, additional studies are needed to investigate PI3K/mTOR inhibitors in cSCC.

### 5.5. Epigenetic Modulators

Cancer prevention, detection, and therapy would likely benefit from controlled manipulation of epigenetic alterations. The perturbation of epigenetic mechanisms that silence tumor suppressor genes and activate oncogenes via altered CpG island methylation patterns, histone modifications, and dysregulation of DNA binding proteins, holds promise. Epigenetic drugs, including two DNA methyltransferase enzyme (DNMT) inhibitors and a deacetylase (HDACs) inhibitor, have been approved by the FDA for cancer treatment. [252]. Various HDAC inhibitors including FK228, SAHA and MS-275, are in phase III clinical trials. Vorinostat, a broad inhibitor of histone deacetylases, belongs to the hydroxymate structural group [253]. Other drugs in this group include Givinostat, Abexinostat, Panobinostat, Belinostat, Remetinostat and Trichostatin A. Vorinostat is currently used as a second-line or concurrent agent in the management of persistent, progressive or recurrent cutaneous T cell lymphoma [254]. Vorinostat has demonstrated activity in human cSCC cell lines and xenograft models, putatively via inhibition of AKT/mTOR signaling and inhibition of EMT by E-cadherin upregulation [255]. A phase II trial of topical Remetinostat was initiated in cSCC (NCT03875859). However, it was terminated after enrolling only 4 patients during the COVID-19 pandemic. Trials of epigenetic therapies using vorinostat in combination with EGFR inhibitors and capecitabine, romidepsin, and 5-azacytidine are ongoing in HNSCC. Mcl-1 levels have been shown to be a key factor in the response to vorinostat, and *FBXW7* mutation is a biomarker for sensitivity to HDAC inhibition [256]. There is also evidence for synergy between treatment with vorinostat and Bcl-2-targeted therapeutics [249]. Epigenetics as biomarkers and therapeutic targets warrant clinical evaluation in cSCC, an important part of advancing these technologies with the development of more specific and effective inhibitors to reduce side-effects, given the wide range of genes and organs epigenetics influence throughout the body. MiRNAs regulate multiple target genes simultaneously and may therefore represent promising therapeutic targets. Development of microRNA therapeutics has accelerated recently, and multiple agents are in preclinical trials.

## 6. Conclusions

**Figure 1:**
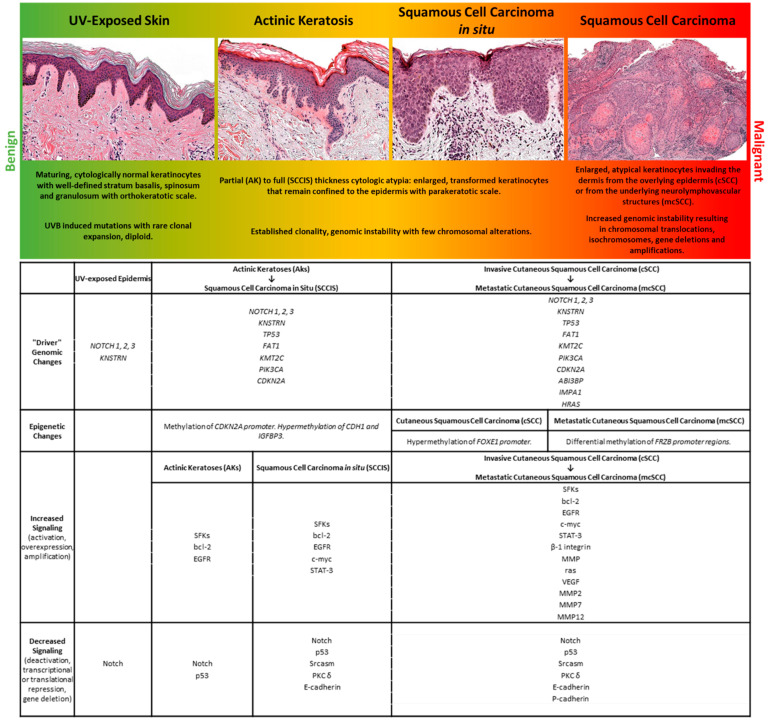
A representation of stages within the spectrum of human cutaneous neoplasia is shown with select histologic and molecular features.

The development, migration, differentiation, and regeneration of the body’s largest organ requires coordination of multiple mechanisms to perform the core skin functions of temperature regulation, protection from a wide range of insults including UV radiation, trauma, pathogens, microorganisms, and toxins, achieved via immunologic surveillance, sensory perception, control of fluid loss, and maintenance of general homeostasis. Similarly, dysregulation of these mechanisms is found in cutaneous tumors, including cSCC. A diagram summarizing the mutations and biological changes occurring at the different stages of UV-induced tumor progression is shown in Figure 1.

Through multi-“omic” studies that dissect the cellular microenvironment of cSCC, we have begun to identify and temporally organize the molecular alterations that underlie the field effect induced by UV irradiation that leads to cSCC [32,34,257]. The progression of oligo-cellular clones of UV irradiated keratinocytes to AKs, SCCIS, and cSCC is mirrored by an accumulation of expression profile changes across a wide array of cell signaling pathways driven by genetic and epigenetic alterations. The sheer number of intracellular and intercellular phenotypic alterations, and an even larger number of mechanisms driving them, is daunting for researchers attempting to understand the biology of this disease. However, this simultaneously offers abundant opportunities for therapeutic intervention and prevention of cSCC. Future studies on the early stages of cSCC tumor development designed to assess the interfacing mechanisms—genetic, epigenetic, metabolomic, and immunologic—that influence the development and progression of lesions to cSCC will offer new opportunities for tailored and combinatorial therapeutic approaches.

## Data Availability

Not applicable.
